# Decision-Making for Risky Gains and Losses among College Students with Internet Gaming Disorder

**DOI:** 10.1371/journal.pone.0116471

**Published:** 2015-01-23

**Authors:** Yuan-Wei Yao, Pin-Ru Chen, Song Li, Ling-Jiao Wang, Jin-Tao Zhang, Sarah W. Yip, Gang Chen, Lin-Yuan Deng, Qin-Xue Liu, Xiao-Yi Fang

**Affiliations:** 1 School of Psychology, Beijing Normal University, Beijing, China; 2 School of Mathematical Science, Beijing Normal University, Beijing, China; 3 State Key Laboratory of Cognitive Neuroscience and Learning and IDG/McGovern Institute for Brain Research, Beijing Normal University, Beijing, China; 4 Center for Collaboration and Innovation in Brain and Learning Sciences, Beijing Normal University, Beijing, China; 5 Department of Psychiatry, Yale University School of Medicine, New Haven, CT, United States of America; 6 Scientific and Statistical Computing Core, National Institute of Mental Health, National Institutes of Health, Department of Health and Human Services, Bethesda, Maryland, United States of America; 7 Faculty of Education, Beijing Normal University, Beijing, China; 8 School of Psychology, Central China Normal University, Wuhan, China; 9 Key Laboratory of Adolescent Cyberpsychology and Behavior (CCNU), Ministry of Education, Wuhan, China; 10 Institute of Developmental Psychology, Beijing Normal University, Beijing, China; 11 Academy of Psychology and Behavior, Tianjin Normal University, Tianjin, China; Erasmus University Rotterdam, NETHERLANDS

## Abstract

Individuals with Internet gaming disorder (IGD) tend to exhibit disadvantageous risky decision-making not only in their real life but also in laboratory tasks. Decision-making is a complex multifaceted function and different cognitive processes are involved in decision-making for gains and losses. However, the relationship between impaired decision-making and gain versus loss processing in the context of IGD is poorly understood. The main aim of the present study was to separately evaluate decision-making for risky gains and losses among college students with IGD using the Cups task. Additionally, we further examined the effects of outcome magnitude and probability level on decision-making related to risky gains and losses respectively. Sixty college students with IGD and 42 matched healthy controls (HCs) participated. Results indicated that IGD subjects exhibited generally greater risk taking tendencies than HCs. In comparison to HCs, IGD subjects made more disadvantageous risky choices in the loss domain (but not in the gain domain). Follow-up analyses indicated that the impairment was associated to insensitivity to changes in outcome magnitude and probability level for risky losses among IGD subjects. In addition, higher Internet addiction severity scores were associated with percentage of disadvantageous risky options in the loss domain. These findings emphasize the effect of insensitivity to losses on disadvantageous decisions under risk in the context of IGD, which has implications for future intervention studies.

## Introduction

Internet gaming disorder (IGD) is defined as excessive and uncontrolled gaming online despite the experience of negative consequences, including insomnia, poor academic performance, and social isolation [1,2]. IGD is increasingly recognized as a mental health issue worldwide [3], as highlighted by its recent including in Section III of the DSM-5 as a topic deserving more future studies [4]. Moreover, since Internet is freely available in campuses, majority of college students play Internet games for recreations, which, however, make them as one of the most susceptible populations to IGD [5,6].

Maladaptive decision-making is one of the key symptoms of addition [7–9]. Previous findings suggest that individuals with substance abuse or dependence have impaired performance on a range of decision-making tasks [10–14]. Recent studies indicate decision-making deficits in IGD. For instance, researchers found that individuals with IGD made more disadvantageous choices on the Game of Dice Task relative to healthy non-playing comparison subjects [15], and that such impairments may be partly a result of a failure to utilize feedback [16]. Evidence also suggests that individuals with Internet addiction are impaired in decision-making under ambiguity measured by the Iowa Gambling Task [17,18]. Neuroimaging studies using other paradigms (e.g., guessing task, probability discounting task) also suggest alterations in neural responses among individuals with IGD during decision-making processes, involving anticipating and processing rewards and punishments [19–21] and evaluating risks [22].

Decision-making is a complex cognitive function, and accumulating evidence suggests that different processes are involved in decision-making for gains and losses [23–26]. Some researchers have found that individuals with addiction-related disorders made significantly more disadvantageous choices primarily in the gain—as compared to loss—domain [27,28], whereas existing data also suggest that insensitivity to losses play an essential role in decision-making deficits among individuals with substance dependence [29,30]. However, the extent to which impaired decision-making among IGD subjects is attributable to alterations in gain versus loss processing remains poorly understood. Separately investigating the characteristics of reward seeking and loss avoidance among individuals with IGD will advance current understanding of the mechanisms underlying decision-making deficits in this population, and may be help in the development of more effective interventions for IGD.

In the current study, we sought to separately evaluate decision-making for gains and losses among college students with IGD. For this purpose, we adopted the Cups task [26], which isolates decision making for the gain and loss domains. In addition, we further sought to examine the effects of two essential components, outcome magnitude and probability level, on decision-making related to risky gains and losses. Based on previous studies [15,16,21], we hypothesized that: (1) IGD subjects, compared to matched healthy controls (HCs) would make significantly more risky choices overall; (2) IGD subjects, in comparison to HCs, would perform worse on risk disadvantageous trials in both the gain and loss domains; (3) decision-making deficits among IGD subjects were associated to insensitivity to outcome magnitude and probability level; and (4) IGD severity scores were positively associated with disadvantageous risky options made on the Cups task.

## Methods

### Ethics Statement

The protocol of this study was approved by the Institutional Review Board of School of Psychology, Beijing Normal University. All participants provided written informed consent before the experiment and received monetary compensation for their participation.

### Participants

A total of 102 college students (60 IGD subjects and 42 HCs) were recruited from universities by online advertisement in Beijing, China. Given the higher prevalence of IGD in men versus women [1,31–33], only male subjects were selected. No participants reported previous experience with illicit drugs (e.g., cocaine) or gambling (including online gambling). Additionally, participants who reported any history of psychiatric or neurological diseases, use of psychotropic medications that affect the central nervous system were excluded from further study.

The diagnosis of IGD was established by weekly Internet gaming time and the Chen Internet addiction scale (CIAS) [34]. The CIAS consists of 26 items, based on a 4-point Likert scale, which evaluates 5 dimensions of Internet addiction: compulsive use, withdrawal, tolerance, problems of Interpersonal relationships, and time management. The reliability and validity of the CIAS among college students has been demonstrated previous [33]. The inclusion criteria for IGD subjects were: (1) scored 67 or higher on CIAS [33,35], (2) spent more time on Internet gaming than any other Internet applications, and (3) spent at least 14 hours a week for at least one year. To further confirm that IGD subjects were addicted to Internet gaming and to rule out the effects of other online activities (especially online gambling) on decision-making, IGD subjects were asked to list first three Internet activities which occupied most of their online time. All of them ranked Internet gaming the first and indicated that they are ‘addicted’ to Internet gaming, but none of them included online gambling or poker games in their lists. The inclusion criteria for HCs were: (1) rating ≤ 50 on CIAS, (2) occasionally Internet gaming (≤ 2 hours per week) or never playing online games in their lifetime.

### The Cups Task

The computerized Chinese version of Cups task was adapted from the original task developed by [26]. The task consists of 54 trials divided into gain and loss domains equally. In each trial, participants were asked to choose between a risky option and a safe option, and the safe option is represented by a single cup and is associated with a 100% probability of either winning or losing 100 yuan. The risky option is represented by 2, 3 or 4 cups and is associated with 50%, 33% or 25% of winning or losing a larger amount of money (possible outcome: 200 yuan, 300 yuan, or 400 yuan). Within each domain, every combination of probability level and outcome level occurs three times, thus gain and loss domains are presented as two separate blocks of 27 random trials. Participants indicated their choice by pressing the left or right button. After each choice, participants were given feedback immediately about the outcome of the trial. The twenty participants who achieved the highest total scores would be provided with an additional bonus.

Based on an independent manipulation of probability level and outcome level, combinations are either: (1) risk advantageous (RA), meaning that the expected value (EV) of risky option is more favorable than that of the safe option; (2) risk disadvantageous (RD), meaning that the EV of risky option is less than that of the safe option; or (3) risk neutral, meaning that the risky and safe options have equal expected values (EQEV).

### Statistical Analysis

Statistical analyses were conducted using SPSS version 20.0 and R version 3.1.0. All tests were two-tailed and the criterion of significance was set at *P* < .05. First, we used independent-sample t-tests to explore group differences in demographic variables. Second, in order to compare the performance of IGD subjects and HCs on the Cups task, we used analyses of variance (ANOVAs) with repeated measurements. In order to explore interaction effects, simple effect analyses were performed. Where Mauchly tests showed violation of the sphericity assumption, Greenhouse-Geisser corrections were used. Post-hoc analyses were conducted using t tests with Bonferroni correction. Third, we separated EV into two components: probability level and outcome magnitude, in order to explore the effect these two components on decision-making for each trial, using the R lmer function of the lme4 library. Finally, to investigate the relationship between Internet addiction severity and decision-making performance of achieving gains and avoiding losses, Pearson’s correlations were used to explore associations between CIAS scores and the percentage of risky choices made during the three EV levels (RA, EQEV, RD) for the gain and loss domains respectively.

## Results

### Demographic Characteristics

As shown in Table 1, the IGD subjects and HCs did not differ in age, average duration of education, and years of lifetime Internet use. Consistent with our inclusion criteria (i.e., CIAS score ≥ 67 for IGA subjects and ≤ 50 for HCs), IGD subjects had significantly higher CIAS scores, *t* (100) = 27.14, *P* < .001. Twenty-two of 42 HCs occasionally played Internet games, however, IGD subjects spent significantly more times on Internet games weekly than HCs, *t* (80) = 15.41, *P* < .001.

**Table 1:** Demographic, Internet use lifetime, the CIAS scores and time spent on IGD subjects and HCs.

	**IGD subjects (n = 60)**	**HCs (n = 42)**	**t value**
	mean ± S.D.	mean ± S.D.	
Age	22.40 ± 2.07	22.38 ± 2.10	0.05
Years of education	15.60 ± 1.81	15.60 ± 1.85	0.01
Internet use lifetime (in years)	9.33 ± 2.79	8.88 ± 2.80	0.81
CIAS scores	79.47 ± 8.77	36.05 ± 6.60	27.14***
Time spent on Internet gaming per week (in hours)	20.97 ± 10.00	1.00 ± 0.49 (n = 22)	15.41***

* *P* < .05;

** *P* < .01;

*** *P* < .001.

S.D. = standard deviation; IGD = Internet gaming disorder; HCs = healthy controls.

Rate of tobacco and alcohol use were low for both groups: three IGD subjects and one HCs reported occasional (less than once a month) cigarette smoking. Nineteen IGD subjects and 12 HCs reported lifetime alcohol use but all with low frequencies (once a week or less), and these rates did not differ between groups, *t* (29) = 1.27, *P* = .216.

### Risk Taking Propensity

Risk taking propensity is a measures of an individual’s tendency to choose the risky option over the safe option at each of the three EV levels (RA, EQEV, RD) calculated separately for the gain and loss domain [36]. We conducted a 2 (domain: gain, loss) × 3 (EV level: RA, EQEV, RD) × 2 (group: IGD subjects, HCs) repeated measures ANOVA. As expected, we observed a main effect of group, *F* (1, 100) = 5.67, *P* = .019, partial η^2^ = .05, indicating that IGD subjects chose more risky options overall than HCs on both gain and loss domain; and a main effect of EV level, *F* (2, 200) = 289.64, *P* < .001, partial η^2^ = .74. Post-hoc analyses showed that participants made more risky options when the EV level was RA than that was RD. The three way interaction between EV level, group and domain did not reach significance, *F* (2, 200) = 1.43, *P* = .242, partial η^2^ = .01. However, we found an EV level × group interaction, *F* (2, 200) = 6.08, *P* = .006, partial η^2^ = .06, and simple effect analysis showed that the significant interaction was mainly due to more risk taking on the RD trails among IGD subjects in comparison to HCs, *F* (2, 99) = 7.54, *P* = .001, partial η^2^ = .13. We also found an significant EV level × domain interaction, *F* (2, 200) = 7.70, *P* = .001, partial η^2^ = .07, and simple effect analysis showed that the participants chose significantly more risky options in the loss domain in comparison to the gain domain on the EQEV (not RA and RD) trials, *F* (1, 100) = 7.57, *P* = .007, partial η^2^ = .07.

**Figure 1:**
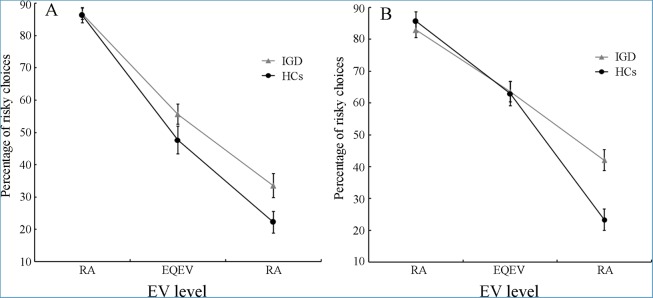
Decision-making performance for IGD subjects and HCs on the Cups task. Mean percentage of risky choices made in (A) the gain and (B) the loss domain, as a function of EV level and group. Error bars reflect standard errors. IGD = Internet gaming disorder; HCs = healthy controls; EV = expected value; RA = risk advantageous; EQEV = equal expected value; RD = risk disadvantageous.

Separate ANOVAs for each domain were further conducted. For the loss domain, in addition to significant main effects of group and EV level, there was a significant interaction effect of EV level × group interaction, *F* (2, 200) = 6.90, *P* = .002, partial η^2^ = .07. Findings from simple effect analyses indicated that IGA subjects made more risky choices than HCs on the RD trials, *F* (1, 100) = 15.11, *P* < .001, partial η^2^ = .13, but did not differ from HCs in the number of risky choices on the RA and EQEV trials (Fig. 1). In contrast, for the gain domain, there was no significant main or interaction effects of group or EV level × group (*P* = .092 and *P* = .138, respectively).

### Sensitivity to Outcome Magnitude and Probability Level

We further separated the EV into two components: outcome magnitude and probability level. In order to examine the effect of these two components on risky decision-making, we conducted logistic hierarchical models using the R lmer function of the lme4 library to take into account trial-by-trial variance in subjects’ risk taking, following the procedure described in a previous study [37]. Two base models respectively for the gain and loss domains included group (0 = HCs, 1 = IGD subjects), probability level (represented probability of winning or losing for risky options: 0.25, 0.33, 0.50), outcome magnitude (2, 3, 4 represented 200, 300, 400 in risky options) and interactions of group × probability level and group × outcome magnitude as fixed-effects predictors, and individual differences in choice as random-effects. The dependent variable was subjects’ choice for each trial (0 = safe option, 1 = risky options).

As shown in table 2, there were significant main effects of probability level and outcome magnitude in both the gain and loss domains. These effects indicated, for both the gain and loss domains, that across both IGD subjects and HCs, subjects took fewer risks as the probability of the risky option became less favorable (main effect of probability level) and that subjects took more risks as the outcome magnitude of the risky option increased (main effect of outcome magnitude).

**Table 2:** Effect of probability level and outcome magnitude on risk taking as a function of domains and groups.

**Parameter**	**Estimate**	**S.E.**	**Wald χ^2^**
Gain domain			
Group	0.27	0.19	2.00
Probability level	1.05	0.08	156.61***
Outcome magnitude	1.12	0.08	179.35***
Group × probability level	-0.19	0.11	3.09
Group × outcome magnitude	-0.18	0.11	3.07
Loss domain			
Group	0.41	0.21	3.88*
Probability level	-1.12	0.08	179.75***
Outcome magnitude	-0.97	0.08	135.30***
Group × probability level	0.29	0.10	8.03**
Group × outcome magnitude	0.41	0.10	15.93***

* *P* < .05;

** *P* < .01;

*** *P* < .001.

S.E. = standard error.

In the gain domain, there were no significant interaction effects between any of the three variables explored. In contrast, in the loss domain there were significant interactions between group × probability level and between group × outcome magnitude, indicating that IGD subjects, relative to HCs, were less likely to adjust their decisions based on probability level and outcome magnitude in the loss domain.

### Correlation between Internet Addiction Severity and Decision-making

Pearson’s correlations were also conducted between CIAS scores and the number of risk choices for the three EV levels (RA, EQEV, RD) separately for the gain and loss domains. In the loss domain, the results indicated that CIAS scores were positively associated and risky choices made on RD trials, r = .22, *P* = .001. The association between CIAS scores were marginally correlated with the number of risky choices in RD trials for the gain domain, r = .19, *P* = 0.056.

## Discussion

To our knowledge, the current study is the first to evaluate risky decision-making among IGD subjects separately for potential losses and gains. Consistent with our first hypothesis, IGD subjects demonstrated generally greater risk taking tendencies on the Cups task, in comparison to HCs. Partially consistent with our second and third hypothesis, IGD subjects made significantly more risky choices than HCs on the RD trials for the loss—but not the gain—domain, and the impairment was associated to insensitivity to changes in outcome magnitude and probability level for risky losses among IGD subjects. Consistent with our fourth hypothesis, correlational analyses further demonstrated significantly positive associations between Internet addiction severity scores and disadvantageous options in the loss domain. Taken together, these data provide further evidence of impairments on decisions under risk among individuals with IGD, and additionally suggest that alterations loss (versus gain) processing may underlie decision-making deficits in this population.

In the loss domain, IGD subjects made more risky decisions on the RD trials relative to HCs, and the trial-by-trial analysis further indicated that IGD subjects were less likely to adjust their decisions based on probability level and outcome magnitude in this domain. These findings are consistent with those from previous studies using similar decision-making tasks and demonstrating impairments in decision-making related to loss avoidance among individuals with substance addictions [38], eating disorders [39], and IGD [16, 19]. One possible explanation for these findings is that, through the repetition of their gaming behaviors, individuals with IGD may more frequently engage in loss-related problem solving, which may render them more tolerant to punishment. In addition, our finding of altered loss-related decision-making is consistent with the clinical presentation of individuals with IGD that they tend to undervalue potential real life negative consequences in order to persist in playing online [2,40,41].

Previous studies have demonstrated elevated disadvantageous risk-taking behaviors in the gain domain among individuals with addiction-related disorders characterized by impairments in impulse control, such as pathological gambling [28] and alcohol dependence [27]. However, neither the results of ANOVA nor trial-by-trial analyses indicated increases in risky decisions on gain trials among IGA subjects. Several possible explanations for these differences exist. Specifically, individuals with pathological gambling exhibit heightened reward responses to monetary versus non-monetary rewards [42], and this may result in greater disadvantageous risk-taking in the gain (versus loss) domain, as has been reported previously [28]. For individuals with alcohol dependence, long lasting and excessive alcohol consumption may alter brain structures and related functions, including key regions in reward processing such as amygdala [43,44]. Evidence indicated that patients with amygdala lesions demonstrated decision-making deficits mainly in the gain domain [26]. Although further research is needed to confirm these hypotheses, the absence of increased risk taking for gains among the IGD subjects could reflect relatively normative processing of monetary rewards (but not losses) in this population. In addition, these findings highlight the importance of assessing different aspects of decision-making across different addiction-related disorders.

Internet addiction severity scores were positive associated with the number of disadvantageous risky choices made on the Cups task, indicating that subjects with higher Internet addiction severity scores made more disadvantageous decisions related to risky losses during RD trials. These findings are in keeping with the previous studies which also reported the preference for disadvantageous risky alternatives was associated with the severity of IGD using similar paradigms, such as the Game of Dice Task [15,16] and the probability discounting task [22]. These findings support the hypothesis that impairments on decision-making related to risky losses are related to the level of Internet addiction severity (i.e., CIAS scores) and may therefore be an appropriate therapeutic target for the treatment of IGD.

Overall, our findings suggest impairments in risky decision-making within the context of loss avoidance among individuals with IGD. Further research is needed to establish the neurobiological basis for these alterations. One hypothesis is that disadvantageous decision-making in the loss domain may relate to alterations in cortico-striatal functioning among individuals with IGD, as has been reported among individuals with behavioral and drug addictions [45–47]. In particular, the insula plays a critical role in the biology of both addiction and decision-making [9,48,49] and is implicated in loss anticipation and avoidance learning [50]. Thus one speculative hypothesis is that impairments in loss avoidance-related decision-making may be related to insular functioning among individuals with IGD.

Several limitations of this study should be noted. First, given that IGD is most prevalent among men [1,32], this study did not include female participants. Thus further studies are needed to assess decision-making for gains and losses among women with IGD. Second, our recruitment of only college students limits the generalizability of our findings. Although college students are one of the most susceptible populations to IGD [5,33], future studies are required to explore the association between the risk-taking for potential gains and losses and IGD within clinical samples. Finally, studies with longitudinal designs are needed in order to investigate whether decision-making alterations are a consequence or a precursor of IGD.

In conclusion, this study is the first to assess decision-making in the gain and loss domains separately among college students with IGA using the Cups task. IGD subjects demonstrated greater risk taking tendencies than HCs. Furthermore, IGD subjects made significantly more risky choices than HCs on the RD trials in the loss but not gain domain, and such impairment was associated with insensitivity to outcome magnitude and probability level related to risky losses. In addition, Internet addiction severity scores were positively associated with disadvantageous risky options made in the loss domain. Taken together, these findings suggest that alterations loss (versus gain) processing may underlie decision-making deficits in this population.

## Supporting Information

S1 File Summarized data. (XLSX)

S2 File Data for trial-by-trial analysis. (XLSX)
